# Patterns of Depressive Symptoms and Social Relating Behaviors Differ Over Time From Other Behavioral Domains for Young People With Down Syndrome

**DOI:** 10.1097/MD.0000000000000710

**Published:** 2015-05-21

**Authors:** Kitty-Rose Foley, Jenny Bourke, Stewart L. Einfeld, Bruce J. Tonge, Peter Jacoby, Helen Leonard

**Affiliations:** From the Telethon Kids Institute, The University of Western Australia, Perth (K-RF, JB, PJ, HL); Department of Developmental Disability Neuropsychiatry, School of Psychiatry, The University of New South Wales (K-RF); Faculty of Health Sciences (SLE); Brain and Mind Research Institute, University of Sydney, Sydney (SLE); Center for Developmental Psychiatry and Psychology, School of Psychology, Psychiatry and Psychological Medicine, Monash University, Melbourne, Australia (BJT).

## Abstract

People with intellectual disabilities are at a higher risk for experiencing behavioral, emotional, and psychiatric problems in comparison with the general population. People with Down syndrome have been reported as experiencing fewer behavioral problems than others with intellectual disability, although still at a greater level than the non-intellectually disabled population, except for depression and Alzheimer disease. The aim of this study was to describe the trajectories of subscales of behavior, including depressive symptoms, communication disturbance, anxiety, disruptiveness, and social relating abilities, for young adults with Down syndrome.

Families of young adults with Down syndrome living in Perth, Western Australia, participated in a questionnaire study over 8 years, 2004 (n = 255), 2009 (n = 191), and 2011 (n = 188). Questionnaires collected information about young person characteristics and family functioning. The parent-completed Developmental Behavior Checklist-Adult (DBC-A) and Developmental Behavior Checklist-Primary Carer Version (DBC-P) were used to measure emotional and behavioral problems. These measures include the following subscales: disruptive, communication and anxiety disturbances, self-absorbed, antisocial, depressive, and social relating.

DBC score declined from 2004 to 2011 reflecting an improvement in behavior in the self-absorbed (coeff −0.011, 95% confidence interval (CI) −0.031, −0.008), anxiety (coef −0.009 95%CI −0.129, −0.006), communication disturbances (coeff −0.008, 95% CI −0.012, −0.005) and disruptive/antisocial behavior (coeff −0.013, 95% CI −0.016, −0.009) subscales. Subscales for depressive symptoms and social relating problems decreased less (coeff −0.003, 95% CI −0.007, −0.0001) (coeff −0.003 95% CI −0.007, 0.001). Young people who were lower functioning were reported as exhibiting significantly more behavioral problems across every subscale when compared with those who were higher functioning.

Behavior of young adults with Down syndrome improves over time but depressive symptoms and social relating behavior problems persist into adulthood. It is possible that those with persistent depressive symptoms are at a high risk for developing depressive illness in adulthood. Identifying young people with Down syndrome who are at risk for developing depression in adult life has implications for prevention and early treatment.

## INTRODUCTION

People with intellectual disability commonly experience behavioral^1^ and mental health problems,^2^ with a prevalence of both ranging from 35% to 40%.^2–4^ These comorbidities negatively influence young people's participation in leisure, recreation, and employment. They can also impact on maternal mental health and family quality of life.^5,6^ Individuals with Down syndrome, the most common chromosomal cause of intellectual disability, have been reported to experience fewer behavioral problems than others with intellectual disability.^7^ In contrast to a representative sample of young people with intellectual disability who were found to have 3 to 4 times the level of clinically significant emotional and behavioral problems than the general population, the risk for those with Down syndrome was less but still twice that of the general population. The problems identified in children with Down syndrome were predominantly oppositional and defiant behaviors with the emergence of depression in adolescents.^7^ Other authors have reported people with Down syndrome to be more susceptible to depression,^8^ with prevalence estimates up to 11% being reported in one study.^9^ As others have identified, there is a clear need to detect and treat psychopathology in adolescents and young adults with Down syndrome.^9,10^

Depression is a debilitating mental health disorder, which is described as experiencing a depressed mood, sleep disturbance, loss of energy, thinking difficulties, thoughts of death/suicide, and feelings of worthlessness.^11^ Identifying depression in young people with intellectual disability presents problems due to difficulties with self-report of symptoms, diminished communication skills, and differences in presentation of psychopathology.^12^ Previous research has described depression in adults with Down syndrome as presenting with symptoms and signs such as withdrawal, mutism, psychomotor retardation, low mood, passivity, decreased appetite, and insomnia.^13,14^ There is a need for research to identify diagnostic markers of depressive symptoms and understand its trajectory in young people with intellectual disability to develop appropriate and timely interventions.

There have only been a few longitudinal studies assessing mental health in people with Down syndrome. Sample sizes have been small and depressive symptoms have not been effectively measured or reported.^15,16^ Cross-sectional studies have reported on problem behaviors in individuals with Down syndrome using different measures. Social problems and thought problems were reported as having the highest problem behavior score in the behavior subscales of the Child Behavior Checklist in a Dutch study involving 322 adolescents with Down syndrome.^10^ Those with Down syndrome were found to exhibit more internalizing behaviors than those without intellectual disability and males were consistently reported as exhibiting more problem behaviors.^10^ Dykens et al^17^ also found that older adolescents with Down syndrome may show decreased externalizing symptoms and slight increases in withdrawal, and internalizing behavior. Recently, researchers examined whether the Strengths and Difficulties questionnaire was appropriate for use in identifying mental health problems in a population of adults with Down syndrome. They found that it had potential for use with people with Down syndrome, yet the sensitivity and specificity were insufficient presenting considerable problems for use in longitudinal research.^18^ Valid and reliable measure of emotional and behavioral problems is key to the usefulness of a longitudinal study, which will accurately identify trajectories of behavior problems for young people with intellectual disability.

The strongest determinants of health, including mental health, for typically developing adolescents, have been identified as income equality and access to education, along with social factors such as personal, family, and community factors. Trajectories of elements of psychopathology and the influence of potential risk and protective factors are not well understood for people with Down syndrome, especially through the transition from child to adolescent to adult.^19^ Adolescence reflects a time of the cumulative effects on behavior from the benefits and/or adversity from the early childhood period, effects on behavior in association with rapid pubertal physical change, and effects experienced due to the maturing brain.^19^ As for the general population, this is a critical time for people with Down syndrome and other intellectual disabilities. During the transition from school to adulthood, young people with intellectual disabilities face multiple challenges by not only learning to manage their personal internal changes through adolescence but also navigating a new and different environment. A recent review of the research on the transition from school to adulthood for young people with intellectual disability highlighted that the scale of the influence of the environment is under-represented.^20^ These multi-faceted challenges may influence the trajectory of psychopathology over this difficult time of transition.

Despite concern about an increased prevalence of depression in adulthood, there is a relative lack of research on the mental health of adolescents with Down syndrome. Thus, the aim of this article was to describe the trajectories of subscales of behavior, including depressive symptoms, communication disturbance, anxiety, disruptiveness, and social relating abilities, for young adults with Down syndrome.

## METHODS

### Participants

Participants for this study were ascertained from the Down syndrome “Needs Opinions Wishes” database in Western Australia, which was established in 1997 with school-aged families identified through the state-wide Disability Services Commission and later the population-based Intellectual Disability Exploring Answers (IDEA) database.^21^ This database has been shown to capture 97% of Down syndrome cases in Western Australia.^22^ Families were invited to participate in the expanded study in 2004 with 500 families (86% of those in the database) able to be contacted and mailed questionnaire packs either directly or through the Disability Services Commission. Comprehensive telephone follow-up was used to improve responses. In this wave, families of children and young people with Down syndrome aged 0 to 25 years were included. A further wave in 2009 included families of young people with Down syndrome aged 15 to 30 years. The main focuses of this wave of data collection were transition-related issues, and therefore did not include those younger than 15 years. In 2011, the age range was 16 to 31 years. The focuses of questionnaires in this wave were post-school participation and day occupation. The only exclusion criterion across all waves of data collection was inability to speak English. Ethical approval for the study was obtained from the Ethics Committee of the Women's and Children's Health Services in Western Australia.

Behavioral information in this population database was gathered from 2004 onwards. Data were collected in the form of questionnaires from families within the database in 2004 (Wave 1, *n* = 255), 2009 (Wave 2, n = 189), and 2011 (Wave 3, n = 188). Of the 255 in Wave 1, 174 were eligible by age for the follow-up studies with questionnaires completed at all 3 waves on 119 (68.4%) and 2 waves on a further 31 (16.7%). Additionally in 2004, a questionnaire was completed on a further 81 young people aged 4 to 11 years who did not meet the age criteria for the transition study in 2009. In 2009, a questionnaire was also completed on 56 young people for whom a questionnaire had not been completed in 2004, and in 2011 on an additional 8 young people for whom it was not completed in 2004 or 2009. In the analysis, data on the 174 and additional 145 (a total of 319 subjects) were used. Questionnaires contained 2 parts: young person characteristics including age, sex, daily activity, functioning in activities of daily living, and behavior; and family characteristics.

### Outcome Measure

Young person emotional and behavioural problems were measured using the Developmental Behavior Checklist Child (96 items) and Adult versions (107 items) (DBC and DBC-A).^1,23^ The DBC was developed specifically for people with intellectual and developmental disability and has been found to be a valid and reliable measure of psychopathology with examples in longitudinal research.^1,23^ Both measures are scored on a 3-point likert scale. Responses can be 0 “Not true as far as you know,” 1 “Somewhat or sometimes true,” or 2 “Very true or often true.” The DBC-A includes 12 items additional to the child version and drops 1 item. The 2 measures can be scored using the 5 DBC subscales and the depressive symptoms subscale, to maximize comparability of the child and adult measures.^1^ The subscales of the DBC include disruptive, self-absorbed, communication disturbance, anxiety, social relating problems, and for this study, the depressive symptoms scores were also calculated as per the DBC-P for wave 1 and DBC-A manual for wave 2 and 3.^24^ The subscale for depressive symptoms in the DBC-A has been shown to have good reliability and validity and was therefore used to measure depressive symptoms in wave 2 and 3, when the young people's age ranges were in the adolescent/adult range.^25^ In wave 1, we used the depressive symptoms subscale of the DBC-P, as this subscale was more applicable to the age range of the cohort.

Young person functioning in activities of daily living was measured using the Index of Social Competence (ISC) in wave 2 and 3 of data collection. The ISC has 3 subdomains describing self-care, community, and communication skills and discriminates well between different levels of ability. In wave 1, functioning was measured using the Functional Independence Measure for Children (WeeFIM).^26^ The WeeFIM has been found to yield reliable results and has been validated in populations of children with Down syndrome.^27,28^

### Statistical Analysis

Each subscale was scored 3 different ways to investigate different elements of emotional and behavioural problems and ensure that the subscales were comparable^29^: mean item score (MIS) reflected overall presence of problem behaviors; intensity index (II) was the proportion of items checked a two, out of all the items checked one or two and measures the severity of problem behaviors; proportion of items checked (PIC) was the proportion of items checked either a one or two and measures the range of problem behaviors.^29^ Problem behavior subscale scores were described in each wave of data collection in terms of MIS, II, and PIC.

Regression models were used to detect linear age trends in subscales of behavior. These models incorporated a random intercept to account for repeated observations on individuals. To further explore the trajectories of change in behavior subscales, regression models with age (categorized into 5 age groups) as the independent variable were used with generalized estimating equations to account for the repeated observations. These analyses were repeated with the cohort stratified into lower and higher functioning in activities of daily living.

## RESULTS

### Participants

The mean age of all participants in wave 1 was 13.9 years (range 3–24 years), wave 2, 21.8 years (15–29 years) and wave 3, 23.5 years (16–31 years). In wave 1, 363 of 500 (72.6%) families returned the questionnaires; however, many families (108/363, 29.8%) did not complete the DBC, due to only being offered the short version of the question (n = 62/363). Response fractions of families who returned questionnaires with sufficient information on behavioral and emotional disturbance of their son/daughter with Down syndrome and age and sex for each wave were as follows: 255 of 301 (84.7%), 191 of 229 (83.4%), and 188 of 223 (84.3%), respectively. The MIS for each of the 6 subscales in 2004 of those eligible for the study in 2009 showed no significant differences between responders and non-responders.

### Change in Behavior From 2004 to 2011 by Subscale

The MIS, II, and PIC of each subscale at each time point are shown in Table 1. Higher scores reflect more problem behaviors. In wave 1, collected in 2004, communication disturbances were the most commonly reported (MIS mean .44 SD 0.31) and the most severe (II mean .29 SD 0.28) problem and were also associated with the largest range (PIC mean 0.34 SD 0.21). The findings relating to prevalence, severity, and range were similar in wave 2 (Table 1). Interestingly, the only subscale in which there was an increase from wave 1 to wave 2 in all areas (MIS, II, and PIC) was the one concerning social relating problem behaviors. However, the only subscale in which there was an increase from wave 2 to wave 3 was the depressive symptoms subscale on average (MIS wave 1 mean 0.25 SD 0.27 to wave 2 mean 0.27 SD 0.28) and in range of problems (PIC wave 1 mean 0.21 SD 0.22; wave 2 mean 0.24 SD 0.21).

**TABLE 1:**
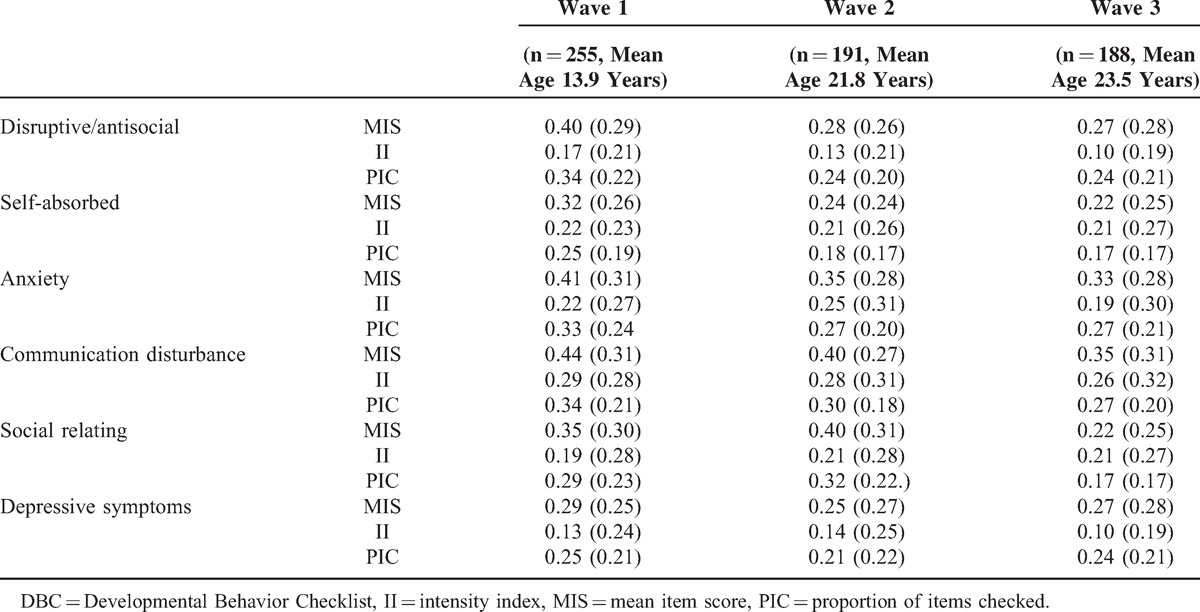
Average DBC Subscale Scores by Wave of Data Collection

**FIGURE 1:**
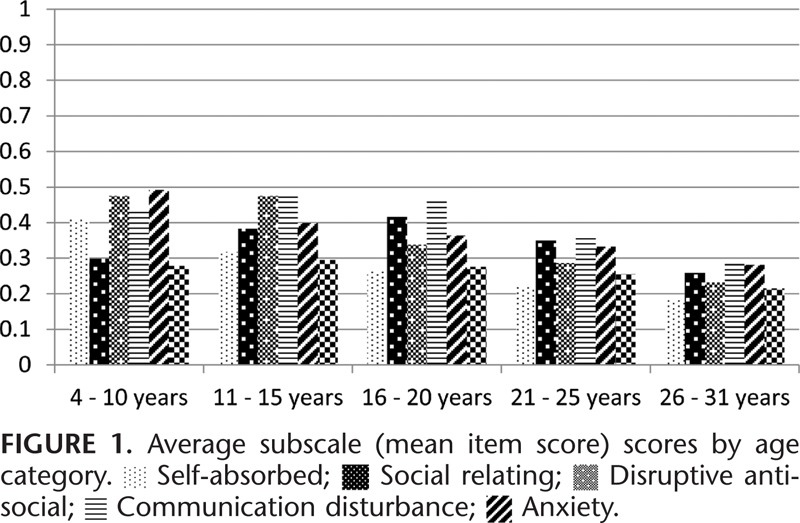


**FIGURE 2:**
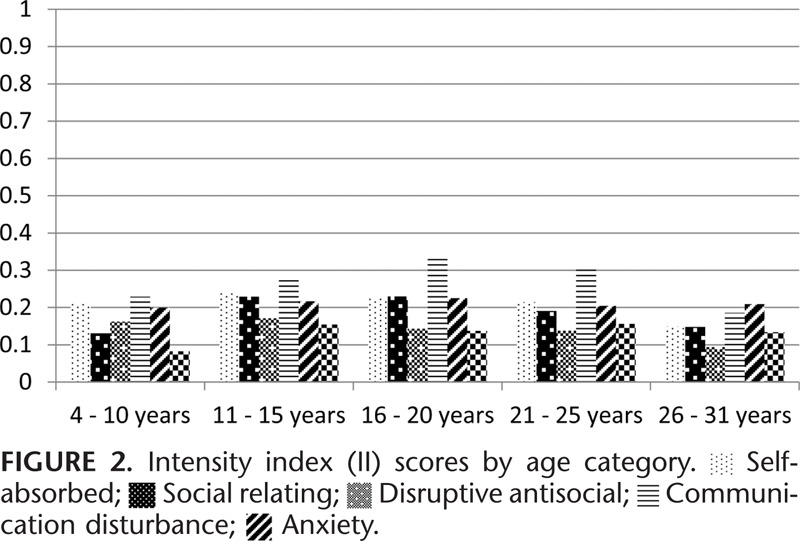


**FIGURE 3:**
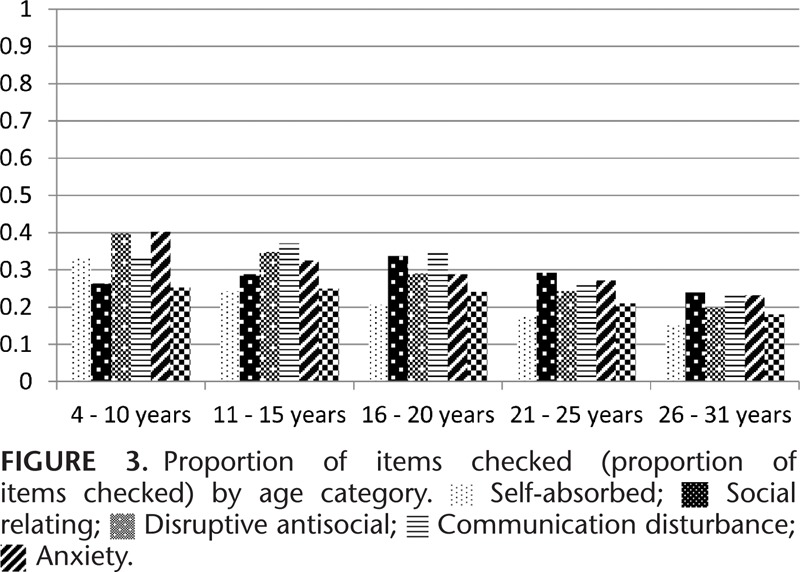


Figure 1 shows average subscale MIS by age category. All data points from all 3 waves of questionnaires are shown on the graph and the correlation between observations at each time point are taken into account. The severity of behavior problems (II) by age category is presented in Figure 2. The severity of behavior problems follows a different trajectory to the MIS with increasing severity into the 16 to 25 years’ age groups followed by a decline in the 26 to 31 years’ age group. Range of behavior problems (PIC) decline steadily across the age categories (Figure 3).

Changes in behavior for each subscale from wave 1 to wave 3 are shown in Table 2. A negative coefficient means that the DBC score declined from 2004 to 2011 reflecting an improvement in behavior. The table is ordered from the subscale that improved the most, to the least. The coefficients reflect a per-point change in the DBC, as these were continuous variables. Disruptive/antisocial behaviors declined the most overall (coeff −0.013, 95% CI −0.016, −0.009), followed by self-absorbed (coeff −0.011, 95% CI −0.031, −0.008), anxiety (coeff −0.009, 95% CI −0.129, −0.006), and communication disturbances (coeff −0.008, 95% CI −0.012, −0.005). The model for social relating (coeff −0.003, 95% CI −0.007, 0.001) and depressive symptoms (coeff −0.003, 95% CI −0.007, −0.0001) showed the smallest decline overtime.

**TABLE 2:**
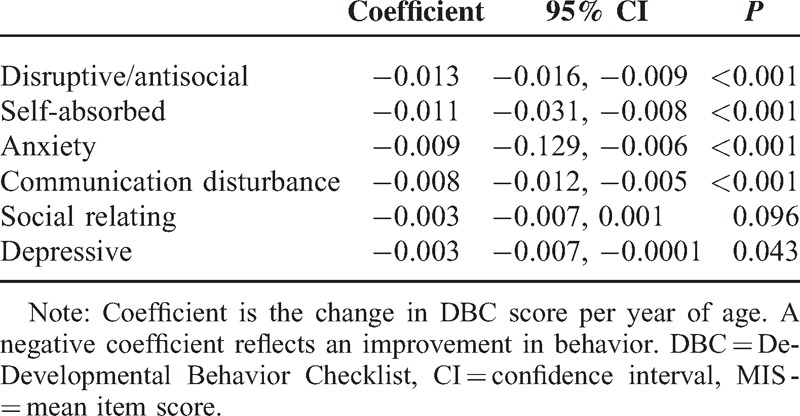
Mean Change in DBC Subscale Scores (MIS) With Age

### Differences by functioning in activities of daily living

**FIGURE 4:**
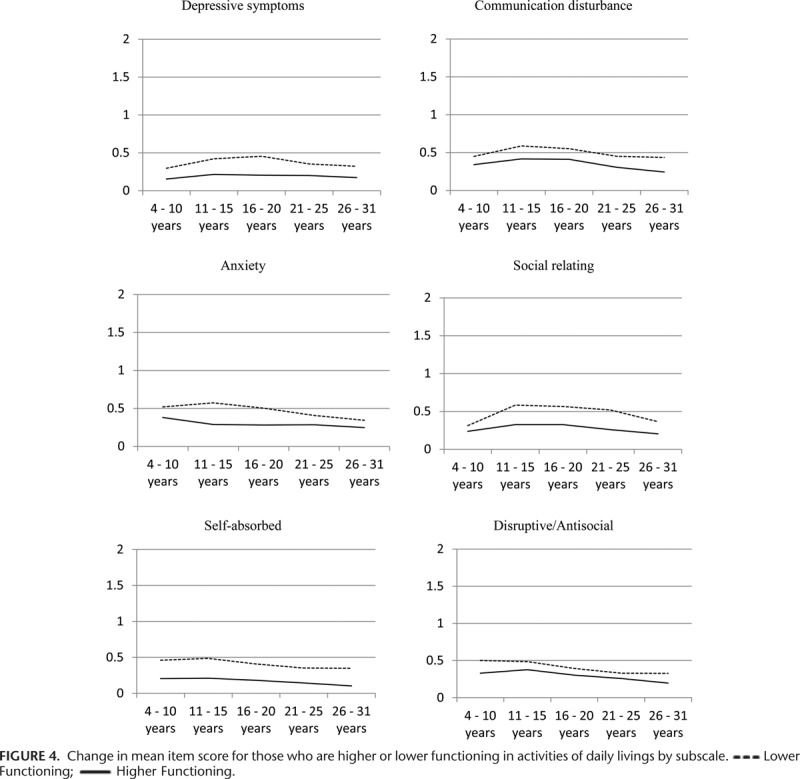


Changes in subscales of behavior problems are presented in Figure 4 and show the difference between those who were reported as having higher or lower functioning in activities of daily living as measured by the WeeFIM (wave 1)^26^ and the Index of Social Competence (wave 2 and 3).^30^ Those who were reported as having lower functioning were reported as exhibiting significantly more problem behaviors across every subscale, including the depressive symptoms subscale. Change overtime varied more across all subscales for those who were reported as having lower functioning than for those who were reported as having higher functioning. The largest differences between those who were reported as having lower or higher functioning were seen in the social relating subscale, and the smallest difference in the disruptive/antisocial subscale.

### Difference by Sex

Table 3 presents change in subscales by sex. A negative coefficient means that females consistently scored, on average, lower than males across all subscales. This reflects that females exhibited fewer behavior problems than males. There was a significant difference in the subscale for self-absorbed behaviors with females reporting on average fewer self-absorbed behaviors than males (coef −0.064, 95% CI −0.114, −0.014).

**TABLE 3:**
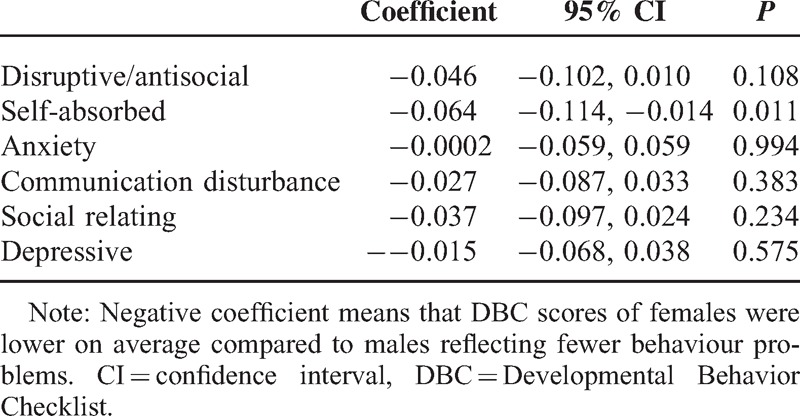
Gender Difference in DBC Subscale Scores Overtime

## DISCUSSION

This study has shown that depressive symptoms and social relating problems persist over time for young people with Down syndrome. These 2 subscales scored the lowest overall in wave 1, reflecting lower levels of depressive symptoms and social relating problems compared with the other subscales, but they did not decline as was seen in the other subscales. The subscale for social relating behaviors increased reflecting more problems for those aged 16 to 20 years and then declined. The subscale for depressive symptoms remained fairly consistent across the different age groups. These findings contribute to the understanding of mental health status across the developmental time periods in people with Down syndrome.

The use of the psychometrically validated measure of emotional and behavioral problems for children and adolescents with intellectual and/or developmental disability, the DBC, was the strength of this study. A limitation is the fact that the data were parent report and we only had 1 source of information. Obtaining data directly from young people with intellectual disability about their mental health status presents challenges, with the need for psychometrically rigorous instruments being highlighted by other authors.^12^ The age range of participants in this study was quite large. We acknowledge that there are important differences between behavior changes across the age ranges in this study and results must be interpreted with caution. Scoring the range and severity of emotional and behavioral problems adds a clinically relevant interpretation of data and allows the results to provide a more focused and specific guide to intervention.^29^ Longitudinal data from a population-based database of young people with Down syndrome allows for analysis, which can reveal important information on changes over time. This is very important information specifically for families and for service providers who can then be guided on how and when to aim specific interventions.

Self-absorbed, anxiety, disruptive, and antisocial behaviors and communication disturbance behaviors improved over time in young people with Down syndrome. Antisocial and communication disturbance behaviors are aspects of externalizing behaviors, which have previously been reported to improve into adolescence for this group.^17^ The internalizing symptoms of self-absorbed behaviors and anxiety improved over time in our study, whereas depressive symptoms, also an internalizing behavior remained persistent. This study did not show evidence of an increase in depressive symptoms during adolescence as is seen in typically developing peers particularly in females.^31^ It was pleasing to see that adolescence and early adult life for those with Down syndrome is a time of relative improvement in mental health compared with childhood. However, the persistence of depressive symptoms from childhood to young adulthood in Down syndrome is concerning. This persistence has also been reported by others.^32^ Perhaps it might indicate that those with persistent depression have a biological Down syndrome vulnerability that makes them more likely to develop major depression in adulthood. Alternatively, persistence in social relating problems might account for at least some of the persistence of depression given some evidence that childhood social relating difficulties have an association with anxiety and depression in young adults with intellectual disability.^33^ Given that the oldest participant was 31 years, it is possible that our cohort has not reached the peak age of incidence of depression. Confirmation of these speculations requires a follow-up study well into adult life.

This persistence of depressive symptoms in young people with Down syndrome has important implications for the ways in which young people with Down syndrome should be supported. Routine screening for potential depressive symptoms and increased awareness among families, carers, and service providers may increase the likelihood these depressive symptoms are identified and can then be addressed. Further research to confirm the persistent presence of depression is required and the development of appropriate, focused mental health interventions for young people with intellectual disabilities.

Young people who were reported as lower functioning compared with being higher functioning were reported exhibiting more problem behaviors across every subscale of the DBC over time. The largest consistent difference was seen in the self-absorbed subscale. Interestingly, the self-absorbed subscale was the only subscale where a sex difference was shown with males exhibiting more self-absorbed problems than females. A study comparing young people with autism and young people with intellectual disability found higher levels of self-absorbed problems in those with autism and in those who had moderate to severe levels of intellectual disability.^34^ Two other studies, 1 from Finland and 1 from Cape Town, also found that those young people with moderate to severe intellectual disability reported higher levels of self-absorbed behaviors compared with those with mild intellectual disability.^35,36^ The South African study, similarly to our study, reported males to be more self-absorbed than females with intellectual disability.

The smallest difference in change over time of problem behaviors for young people with Down syndrome who are higher compared with lower functioning was in the disruptive/antisocial subscale. This subscale also decreased the most over time, reflecting an improvement in disruptive/antisocial behaviors. A longitudinal Australian study found that their participants with mild intellectual disability scored significantly higher than those with severe or profound intellectual disability in disruptive/antisocial behaviors, yet they also showed larger decreases over time.^1^ Disruptive behaviors included noisy, abusive, impulsive, manipulative, and bossy behaviors and have significant clinical implications. Disruptive behaviors have been associated with heightened parent burden, out-of-home placement of children with intellectual disability, and teacher burn-out.^37–39^ The large decrease in disruptive antisocial behaviors over time for young people with Down syndrome is positive, yet the small differences between those who are lower or higher functioning is interesting and warrants further investigation.

The change in severity of behavior problems over the different age categories offers intriguing information about the potential impact the problem behaviors have at different life stages. There were more problem behaviors according to the average problem behavior scores during childhood; however, these behaviors were more severe during adolescence and early adulthood than they were during childhood. Increased intensity of problem behaviors could potentially result in more severe impacts on both the young person's and family functioning. Research has investigated motivations for different problem behaviors and has identified factors such as attention, sensory stimulation, pain reduction, social escape, and tangible reinforcement as purposes for problem behaviors.^40^ However, research has not investigated how the severity of problem behaviors may influence different outcomes.
